# Hepatitis B serology testing and vaccination for Gambian healthcare workers: A pilot study

**DOI:** 10.4102/jphia.v15i1.489

**Published:** 2024-07-24

**Authors:** Buba Manjang, Ebrima Keita, Sheikh Omar Bittaye, Bubacarr Jallow, Sambou Mbye, Abdoulie B. Badjie, Ibrahim Touray, Lamin Bojang, Saydiba Tamba, Lamin Kebbeh, Lamin M. Bojang, Sanna Kanyi, Modou Lamin Sanneh, Njaga Ceesay, Joanna M. Gaitens, Hanna M. LeBuhn, Melissa A. McDiarmid

**Affiliations:** 1 Department of Public Health, Faculty of Medicine and Allied Health Sciences, University of The Gambia, Banjul, Gambia; 2 Department of Public Health, Faculty of Medicine and Allied Health Sciences, National University of Malaysia, Kuala Lumpur, Malaysia; 3 Department of Public Health, Faculty of Medicine and Allied Health Sciences, University of Birmingham, Birmingham, United Kingdom; 4 Directorate of Public Health Services, Occupational Health and Safety Unit, Ministry of Health, Banjul, Gambia; 5 Department of Internal Medicine, Faculty of Medicine and Allied Health Sciences, University of The Gambia, Banjul, Gambia; 6 Department of Internal Medicine, Edward Francis Small Teaching Hospital, Banjul, Gambia; 7 Directorate of Public Health Services, Ministry of Health, Banjul, Gambia; 8 Directorate of Public Health Services, Environmental Health Unit, Ministry of Health, Banjul, Gambia; 9 Department of Internal Medicine, Faculty of Internal Medicine, Edward Francis Small Teaching Hospital, Banjul, Gambia; 10 Department of Laboratory Medicine, Faculty of Pathology, Edward Francis Small Teaching Hospital, Banjul, Gambia; 11 National Public Health Laboratories, Ministry of Health, Banjul, Gambia; 12 Department of Medicine, University of Maryland, Baltimore, United States of America

**Keywords:** prevalence, serology testing, hepatitis B virus, healthcare workers, occupational health

## Abstract

**Background:**

Hepatitis B infection is a significant global health threat contributing to healthcare worker (HCW) harm, threatening already precarious health systems.

**Aim:**

To document self-reported hepatitis B vaccination history and serology results.

**Setting:**

A select group of high-risk HCWs in a tertiary care hospital in Banjul, the Gambia.

**Methods:**

This was a cross-sectional pilot study conducted from 12 June 2023 to 16 June 2023. Participants were HCWs at high risk for blood exposure who completed a health history interview prior to serology testing for hepatitis B surface antigen (HBsAg) and hepatitis B surface antibody (anti-HBs) and vaccination.

**Results:**

The pilot study enrolled 70 HCWs who were primarily female (*n* = 44; 62.9%). The majority of the participants, 43 (61.4%) reported having received at least one dose of the hepatitis B vaccine in the past. The overall prevalence of HBsAg positivity in this study was 4.3% (95% confidence interval [CI]: 1.5–11.9), all in older participants. Importantly, 60.0% (95% CI: 48.3–70.7) of participants had no anti-HBs detected.

**Conclusion:**

This pilot study documents a higher prevalence of hepatitis B infection among older workers and the lack of anti-HBs across the majority of participants. This suggests a serious vulnerability for the individual health worker and indicates the need for a wider screening and vaccination campaign to assess the risk across the Gambian health workforce.

**Contribution:**

This pilot study provides the first evidence to support a wider assessment of hepatitis B serology status of Gambian health workers to gauge the need for a broader vaccine campaign.

## Introduction

### Background

Hepatitis B infection is a significant global health threat that contributes to the loss of healthcare workers (HCWs) and puts the health workforce at considerable risk.^1^ According to the World Health Organization (WHO), viral hepatitis is responsible for approximately 1.34 million deaths annually.^2^ Healthcare workers have a four-fold increased risk relative to the general population for exposure to hepatitis B virus (HBV) from infected patients.^1,3^ As a result of this significant public health challenge, the WHO Assembly adopted the first global health sector strategy on viral hepatitis in 2016 to protect the global health workforce.

In Africa, HBV is estimated to affect 15% – 20% of the population.^4^ In a low- and middle-income country (LMIC) like the Gambia, HBV prevalence varies ranging from 13% among blood donors,^5^ 9% among pregnant women,^6^ and between 8 and 17% among human immunodeficiency virus (HIV)-infected individuals.^5^ Although the prevalence of HBV varies in the Gambian population and is relatively high, HCWs are not systematically vaccinated against HBV due to a history of infant vaccine campaigns in the last 30 years and several cultural, political and socioeconomic factors.

The loss of HCWs during both the Ebola crisis in West Africa and the coronavirus disease 2019 (COVID-19) pandemic^7,8,9^ has demonstrated how indispensable HCWs are to a functioning and resilient health system. Healthcare worker protection must therefore become more strongly prioritised in countries where health systems are already fragile.^10^

### Health worker protections in the Gambia

The Gambian Ministry of Health (MoH), with support from the University of Maryland, Baltimore’s (UMB) WHO Collaborating Centre for Occupational Health and the University of the Gambia School of Medicine and Allied Health Sciences has collaborated since 2014 through a series of multi-day trainings, hospital and clinic site visits and key informant consultations to build capacity in basic occupational health services for health workers. These activities formed the basis of a National Occupational Health and Safety Policy for Healthcare Workers completed in 2018 and validated in 2020. One of its priorities was prevention of blood-borne hazards (e.g., hepatitis B, C and HIV) in the health workforce. Following the validation, an implementation plan was drafted. Due to the high prevalence of HBV in the general population in the Gambia, there was a concern for health worker risk, given that systematic vaccination of health workers had not been standardised in the Gambia. However, the younger population had likely received part or all of the vaccine series as infants. To clarify the need for vaccination, the decision was taken to assess hepatitis B serology markers in a pilot study of health workers.

### History of childhood vaccines in the Gambia

Maintaining high immunisation coverage is a key component in reducing morbidity and mortality from vaccine-preventable diseases. In 1974, the WHO launched the Expanded Programme on Immunization (EPI) to make vaccines available to all children.^11^ Five years later, the EPI was established in the Gambia to target childhood diseases, including hepatitis B. From 1986 to 1990, the Gambia launched the nationwide Gambia Hepatitis Intervention Study (GHIS) which targeted infant HBV vaccination as part of the EPI.^12^ The objective of the GHIS study was to evaluate the protective effectiveness of infant HBV vaccination on the incidence of hepatocellular carcinoma (HCC) in adulthood.^13^ While infant vaccination was included in the GHIS, coverage may have been variable. In addition, the three-dose series means that a sizable number of eligible children may not be fully immunised. Final results from the study were estimated to take 30–35 years and complete elimination of infection was expected to take 20–30 years.^13^ When the trial finished in 1990, the national infant hepatitis B vaccination programme replaced GHIS. Given initial positive results in LMICs globally, the WHO recommended that all member states include the hepatitis B vaccine in their national childhood immunisation services by 1997.^14^

### Waning immunity

While the Gambia has worked to expand childhood vaccination coverage, and studies have shown that the full three-dose primary hepatitis B vaccination series provides long-term immunity, it may not provide lifelong protection as immunity has been shown to wane over time.^15,16^ A previous study analysing HBV immunity 15 years post-immunisation concluded that one or more boosters are needed to protect individuals from breakthrough infections.^17^ Another study looked at serologic hepatitis B immunity in HCWs and found that 29% of workers who were vaccinated against hepatitis B showed no serologic evidence of hepatitis B immunity.^18^ The lack of response in a percentage of HCWs means that many are still at risk for infection.

### Barriers to hepatitis B vaccine uptake

In addition to waning immunity, the three-dose vaccine schedule puts a strain on families that experience travel-related barriers during infant vaccine campaigns, meaning that some children may not be fully covered. Barriers to vaccination in adult HCWs include financial costs of vaccine distribution, lack of hospital policy, low-risk perception, fear of side effects, lack of time, insufficient cold-chain storage and lack of trained community health workers.^15,16,19,20,21^ Furthermore, a lack of awareness of the vaccine’s effectiveness contributes to inadequate vaccine uptake.^22^ The combination of each of these barriers causes vaccination coverage to plateau.

### Interest in high-risk adults

Although historically the WHO has supported vaccine campaigns for many vaccine-preventable diseases (VPDs), their focus has been almost exclusively on the paediatric population.^23^ Recently, however, the WHO has begun to expand its focus on the immunisation of vulnerable adult populations. Because HCWs are frequently exposed to infectious patients, they are considered an especially vulnerable adult population. In 2022, the WHO released an implementation guide for the vaccination of HCWs that outlined the latest recommendations and programmatic considerations for the vaccination of HCWs.^24^ Specific vaccination recommendations include hepatitis B, as well as influenza, measles, mumps, rubella, pertussis and varicella. The guide highlights the need to integrate HCW vaccination into existing occupational health and safety policies and suggests that, as part of a national comprehensive viral hepatitis response, countries may consider establishing a hepatitis B testing and vaccination approach for health workers at no cost to the employee. This report describes initial efforts undertaken to include hepatitis B serology testing and vaccination in the occupational health programme for the health workforce in the Gambia, West Africa.

### Study objective

The objective of this pilot study was to document self-reported hepatitis B vaccination history and hepatitis B serology results in a select group of high-risk HCWs in a tertiary care hospital in the Gambia.

## Research methods and design

This descriptive, cross-sectional pilot study was conducted at Edward Francis Small Teaching Hospital (EFSTH) in Banjul from 12 June 2023 to 16 June 2023. The EFSTH is the only tertiary hospital in the Gambia. The hospital serves as the primary referral centre for the nation and sees patients from across the country.

All HCWs from the main laboratory, the dialysis unit, and the labour and delivery unit were invited to participate in this pilot study as they were likely to be at increased risk for blood and body fluid exposure because of the nature of their job duties. To publicise the effort and promote participation, two of the senior investigators met with the hospital administration prior to the planned start date to explain the project background and protocol. The hospital, through the human resource department, sent a memo to each of the hospital department heads. The memo was then shared in the WhatsApp hospital communication groups of the different departments to inform them about the study and the schedule date, time and place.

The screening team was composed of principal investigators, clinical investigators, counsellors, data collectors, laboratory staff, vaccinators and nurses. Before the screening, the team met and determined roles and responsibilities of staff which were reiterated each morning before the start of the screening day. Once the team was set up each morning, the principal investigator and the clinical investigator visited respective work unit locations or offices to briefly refresh potential participants about the pilot and invite them for screening. This was done daily to serve as a gentle reminder for those yet to be screened.

**FIGURE 1:**
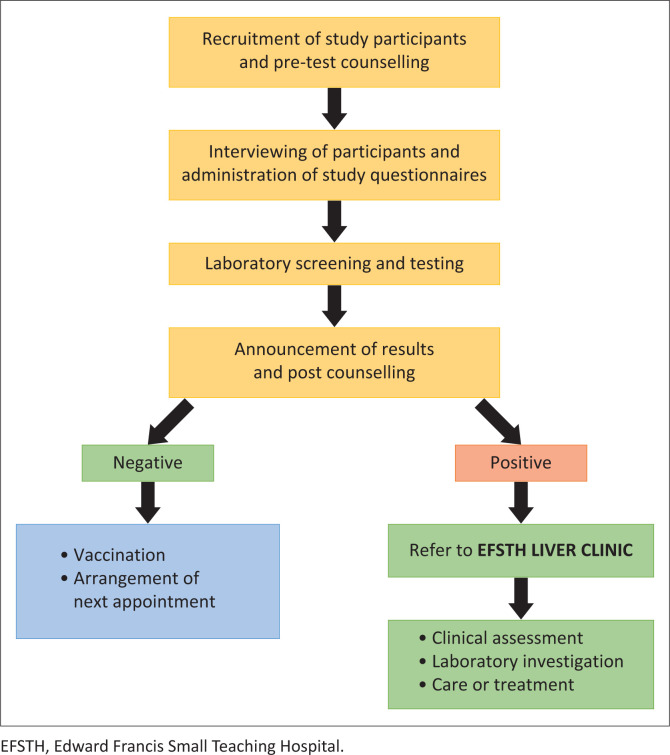
Pilot hepatitis B serology testing and vaccination of health workers in Edward Francis Small Teaching Hospital operational methodology at the screening site.

The study participants were first seen at the counselling unit for pre-test counselling and to obtain written informed consent (see Figure 1). After a participant consented, a study number was allocated, which became their study identifier to maintain confidentiality. The participants were then sent to the data collection room. The data collectors then administered the study questionnaire, which included questions about demographics, vaccination history and clinical department. Country of origin was also asked because the Gambia was an early adopter of the infant hepatitis B vaccine, permitting participant age to be a likely proxy for previous vaccine. Participants were then sent to the laboratory with a form where 2 mL of blood was collected in an ethylenediaminetetraacetic acid (EDTA) tube. The sample was spun for 5 min at 5000 revolutions per minute (rpm) and the plasma was used for rapid hepatitis B surface antigen (HBsAg) (Rapid Card Instatest, Diagnostic Automation/Cortez Diagnostics, Woodland Hills, California, United States) and anti-HBs tests (Rapid Response HepBs Ab test cassettes, BTNX Inc, Ontario, Canada) which takes about 15 min. The rapid HBsAg test has a relative sensitivity of > 99.5%, relative specificity of 99.6%, and accuracy of 99.0% as reported by the manufacturer. The anti-HBs rapid test has a reported relative sensitivity of > 99.9%, relative specificity of 99.4% and accuracy of 99.6%.

Results were recorded in the form, sealed and handed over to the counsellor in the counselling room for post-test counselling. Those with negative HBsAg results were then referred to the vaccination room for the first adult hepatitis B vaccination dose. They were also scheduled for the subsequent 2nd and 3rd doses. Those with positive HBsAg results were referred to the EFSTH liver clinic for liver assessment (Figure 1). Due to delays in receiving anti-HBs test results and concerns about waning immunity even if an individual was vaccinated previously, all participants with a negative HBsAg result were offered the initial dose of the hepatitis B vaccine.

### Linkage to care

Among HCWs who tested positive for HBsAg, linkage to care involved visiting the EFSTH liver clinic at least once after screening for a liver disease assessment. As part of the assessment, those HBsAg-positive participants had an ultrasound scan performed by the clinical investigator. Blood samples were also collected for haematology, biochemistry and hepatitis B virus deoxyribonucleic acid (HBV DNA).

### Data analysis

Data were entered using a tablet and then imported and analysed using Kobo Collect. Simple proportions were calculated for discrete demographic characteristics and outcome variables. For the main outcome variables (proportions of positive and negative serology test results), 95% confidence intervals (CIs) were also determined. In analysing the serology results, the participants were also divided into two groups based on age. The first group includes younger HCWs (≤ 33 years) who were born after the introduction of the nationwide hepatitis B vaccine into the expanded programme of immunisation in 1990. The second group, the older cohort (> 33 years), were born before the introduction of the nationwide hepatitis B vaccination into the expanded programme of immunisation.

### Ethical considerations

Ethical clearance to conduct this study was obtained from the Edward Francis Small Teaching Hospital Research Ethics Committee (REC) (No. EFSTH_REC_2023_050).

## Results

### Demographic characteristics of the participants

A total of 70 HCWs in EFSTH were enrolled in this pilot study. Most of the participants were female (*n* = 44; 62.9%) and Gambian (*n* = 67; 95.7%). The median age of the participants was 35 years (interquartile range [IQR]: 31–41). The majority of the participants, 43 (61.4%) also reported having taken at least one dose of the hepatitis B vaccine in the past (Table 1). Almost one-third (22; 31.4%) of the participants attained a bachelor’s degree and most (*n* = 43; 61.4%) of them worked in the clinical laboratory (Table 1).

**TABLE 1:** Demographic characteristics of participants (*n* = 70).

Variable	*n*	%
**Gender**		
Male	26	37.1
Female	44	62.9
**Age groups (Years)**		
≤ 33	28	40.0
> 33	42	60.0
**Nationality**		
Gambian	67	95.7
Non-Gambian	3	4.3
**Education level**		
Less than high school	27	38.6
High school diploma	7	10.0
Non-degree or certificate programme	9	12.9
Bachelor’s degree	22	31.4
Master’s degree	3	4.3
Professional or doctorate degree	2	2.9
**Occupational sites of participants**		
Clinical laboratory	43	61.4
Dialysis	12	17.1
Labour and delivery	10	14.3
Administrative office	4	5.7
Intensive or critical care	1	1.4
**Previously taken at least a dose of hepatitis B vaccine**		
Yes	43	61.4
No	24	34.3
Not sure	3	4.3

### Prevalence of hepatitis B infection

The prevalence of hepatitis B infection among the HCWs in this pilot study was 3 (4.3%, 95% CI: 1.5–11.9). All the 3 positive results were in the older cohort (7.1%). There were no positive antigen results in the younger cohort (Table 2).

**TABLE 2:** Prevalence of hepatitis B surface antigen.

Variable	*n*	%	95% CI
**Hepatitis B surface antigen (*n* = 70)**			
Positive	3	4.3	1.5–11.9
Negative	67	95.7	88.1–98.5
**Age groups (years)**			
Younger cohort ≤ 33 years (*n* = 28)			
Positive	0	0.0	-
Negative	28	100.0	-
Older cohort > 33 years (*n* = 42)			
Positive	3	7.1	-
Negative	39	92.9	-

CI, confidence interval.

*Prevalence of anti-HBs*. Of the 70 participants included in the study, only 28 (40.0%, 95% CI: 29.6–51.7) tested positive for anti-HBs. Hepatitis B antibody testing was negative in 42 (60.0%, 95% CI: 48.3–70.7) participants (Table 3). Out of the 26 participants who were fully vaccinated, 19 (73.1%) tested positive for anti-HBs and 7 (26.9%) tested negative; while out of the 17 participants who were partially vaccinated, 5 (29.4%) tested positive for anti-HBs and 12 (70.6%) tested negative. Of those who were not vaccinated, 21 (87.5%) tested negative for anti-HBs, but 3 (12.5%) of the never-vaccinated participants tested positive for anti-HBs. As vaccination history was self-reported, it is possible that the three individuals who reported never being vaccinated were unaware of the own vaccine status. It is also possible that these individuals may have recovered from a previous hepatitis B infection, especially since two of the three individuals were born before the Gambian infant vaccination programme was initiated. Of the three participants who were not sure of their vaccination status, 1 (33.3%) tested positive and 2 (66.7%) tested negative for anti-HBs.

**TABLE 3:** Hepatitis B antibody test results.

Self-reported vaccination status	Antibody test results		
	Positive	Negative	Total
Fully vaccinated (3 doses)	19	7	26
Partially vaccinated (1 or 2 doses)	5	12	17
Not vaccinated	3	21	24
Not sure of status	1	2	3

**Total (*n*)**	**28**	**42**	**70**

Note: Total antibody test results: Positive – 40.0% (95% CI: 29.3, 51.7); Negative – 60.0% (95% CI: 48.3, 70.7); Total – 100.0%.

CI, confidence interval.

### Vaccination after screening

Of the total, 65 (86.7%) participants, who all tested negative for HBsAg were vaccinated, and 5 (3.3%) were not vaccinated. Two of those who were not vaccinated tested negative for HBsAg and three tested positive for HBsAg. Of the three testing positive for HBsAg, two reported never being vaccinated and one reported being fully vaccinated.

## Discussion

Vaccinating health workers is a cost-effective investment and a prerequisite for building a robust health workforce. As such, WHO recommends the development and implementation of national policies on vaccination of health workers.^24^ For hepatitis B, such a plan would include systematic assessment of serology markers for new workers, the provision of vaccine at no cost to the worker, if needed, a confidential health record system, and linkage to care for those who are already infected also at no cost, with on-going follow-up and treatment as needed.

These actions bolster the stability and resilience of emerging health systems and directly impact the achievement of the United Nations Sustainable Development Goals (SDGs) targets.^25^ The Gambian Health Ministry has been working towards these goals for more than 10 years. Although the Gambia has been a location of infant hepatitis B vaccination trials since the late 1980s, with the lack of documentation, barriers to vaccine coverage, and the likely waning immunity over the 30 ensuing years, the immune status of the current health workforce is unknown.

Examining first, those likely at highest risk for exposure to blood and body fluids, this pilot found a prevalence of hepatitis B infection of 4.3%. There were no positive HBsAg findings among the younger cohort. The prevalence in the older cohort was 7.1%. Although our population was not intended to be a representative sample, this result is similar to other studies done in the Gambia among the adult population which showed an 8.2% prevalence of hepatitis B.^26^ All of the hepatitis B-positive cases in this pilot were in the older cohort. These HCWs were born before 1990 when nationwide HBV vaccination was introduced into the Gambia’s EPI. Identifying these cases prior to presentation of clinical symptoms permits early treatment and may result in better health outcomes allowing them to continue to work.^27^

This study also showed that 60.0% of the HCWs tested were negative for anti-HBs. This finding demonstrates that a majority of HCWs in this pilot study were not protected against HBV infection, even as they are at high risk of exposure to potentially infectious patients. While these results may not be representative of the Gambian health workforce, it is the only estimate available and suggests the need to extend this serology testing nationally and vaccinate where needed.

### Limitations

Limitations of this pilot include the use of self-reported data for the history of vaccination and the use of age as a proxy for the likely vaccinated and likely unvaccinated participant sub-groups. We also used rapid tests for antigen and antibody determinations which may not be as sensitive and reliable as laboratory-based enzyme-linked immunosorbent assay (ELISA) and immunoassays. However, the products that we used had very high sensitivity and specificity and the benefit of the rapid tests at the point of care permitted valuable on-site clinical decision-making.

The significant prevalence of HBsAg-positive participants and the low prevalence of anti-HBs protection in this small pilot population may not be representative of the risk in the larger Gambian health workforce. The participants in this pilot were deliberately selected due to their high risk of exposure to blood and other potentially infections body fluids, and thus may represent a worst case. However, the duty station in Banjul may have afforded more opportunities for episodic vaccination than may have occurred in more remote areas.

## Conclusion

This pilot study documents the lack of hepatitis B antibody protection in a large proportion of the HCW participants. These results emphasise the need to assess the larger HCW population in the already challenged Gambian health system to ensure protection against this vaccine-preventable disease. Thus, there is an urgent need for implementing robust policies for systematic HBV screening and vaccination among HCWs throughout the Gambia. This will provide benefits at both the individual and health systems strengthening levels.
